# Evaluation of mhGAP training for primary healthcare workers in Mulanje, Malawi: a quasi-experimental and time series study

**DOI:** 10.1186/s13033-020-0337-0

**Published:** 2020-01-20

**Authors:** Demoubly Kokota, Crick Lund, Jennifer Ahrens, Erica Breuer, Sheila Gilfillan

**Affiliations:** 1 Department of Mental Health, University of Malawi, College of Medicine, Chichiri, Blantyre 3, P/Bag 360, Malawi; 2 Alan J Flisher Centre for Public Mental Health, Department of Psychiatry and Mental Health, University of Cape Town, 46 Sawkins Road, Rondebosch, Cape Town, 7700 South Africa; 3 Centre for Global Mental Health, King’s Global Health Institute, Health Services and Population Research Department, Institute of Psychiatry, Psychology and Neuroscience, King’s College London, London, UK; 4 Mile End Hospital, Bancroft Road, London, E1 4DG UK; 5 Department Mental Health, University of Malawi, College of Medicine, Chichiri, Blantyre 3, P/Bag 360, Malawi; 6 University of Newcastle, University Drive, Callaghan NSW, New Castle, 2308, Australia; 7 Royal Edinburgh Hospital, Morningside Park, Edinburgh, EH10 5HF UK; 8 Department of Mental Health, University of Malawi, College of Medicine, Chichiri, P/Bag 360, Blantyre 3, Malawi

**Keywords:** mhGAP, Malawi, Effectiveness, Implementation, Training package, Supervision

## Abstract

**Background:**

There has been a growing global movement championed by the World Health Organization (WHO) to integrate mental health into primary health care as the most effective way of reducing the mental health treatment gap. This study aimed to investigate the impact of WHO Mental Health Gap Action Programme (mhGAP) training and supervision on primary health workers’ knowledge, confidence, attitudes and detection rate of major mental disorders in Mulanje, Malawi.

**Method:**

The study used a quasi-experimental method (single cohort pre- and post-measures) with an interrupted time-series design. A 2 day mhGAP training was delivered to 43 primary healthcare workers (PHWs) working in 18 primary care clinics serving the entire population of Mulanje, Malawi (population 684,107). Modules covered were psychosis, moderate-severe depression, and alcohol & substance use disorders. The PHWs completed pre and post-tests to assess knowledge, confidence and attitudes. Number of diagnosed cases was obtained from clinic registers for 5 months prior to and 7 months following training. Data was analyzed using mean scores, t-test, one-way analysis of variance and linear regression.

**Results:**

The mean knowledge score increased significantly from 11.8 (SD: 0.33) before training to 15.1 (SD: 0.38) immediately after training; t (42) = 7.79, p < 0.01. Similarly, mean knowledge score was significantly higher 6 months post training at 13.9 (SD: 2.52) compared to before; t (42) = 4.57, p < 0.01. The mean confidence score also increased significantly from 39.9 (SD: 7.68) before training to 49.6 (SD: 06.14) immediately after training; t (84) = 8.43, p < 0.01. It was also significantly higher 6 months post training 46.8, (SD: 6.03) compared to before; t (84) = 6.60, p < 0.01. One-way analysis of variance showed no significant difference in mean scores on all four components of the scale used to measure attitudes. A significant positive change in the trend in mental health service utilization after the intervention was demonstrated using a segmented linear regression (β = 2.43 (95% CI 1.02; 3.83) as compared to before (β = − 0.22 (95% CI − 2.67; 2.23) and immediately after (β = 1.63 (95% CI − 7.31; 10.57).

**Conclusion:**

The findings of this study add to the growing evidence for policy makers of the effectiveness of mhGAP training and supervision in a resource-constrained country.

## Background

The treatment gap for mental and substance use disorders in Africa and other low-income countries (LICs) is between 70 and 90% [1]. This is despite growing evidence that it is possible to provide cost effective interventions for many mental disorders in low-income settings [2]. One of the reasons for the high treatment gap in LICs is the lack of human resources for mental health. According to the World Health Organization’s Mental Health Atlas 2017, there are 11.9 psychiatrists per 100,000 population in high-income countries (HICs) compared to less than 0.1 per 100,000 population in LICs countries [3]. Moreover, there are only 0.3 psychiatric nurses per 100,000 population in LICs as compared to 23.5 per 100,000 in HICs [3]. Task-shifting to non-specialists is one of the potential solutions to overcome this human resource problem [4].

Malawi is one of the most poorly resourced countries in Africa in relation to mental health. There are only three main specialist psychiatric institutions located in each of its three regions with a total number of 400 psychiatric beds (2.56 beds per 100,000 population) [3]. These institutions are Zomba Mental Hospital (ZMH) in the south, Bwaila Psychiatric Unit in the centre and St John of God in the north. Currently, Malawi has only three psychiatrists and four psychologists for a population of 18.6 million people. ZMH has only 1 occupational therapist and no professional social worker. Trained psychiatric nurses find that once in post, the majority of their time is often spent on other urgent clinical activities, with few able to work full time providing mental health care [5]. The few health workers who are able to provide mental health care in primary care settings are usually hindered by medication shortages and transportation problems.

There has been a growing global movement to integrate mental health into primary health care [2, 6]. This is regarded as the most effective way of addressing the global burden of mental and substance use disorders and reducing the treatment gap for people with mental disorders in resource constrained settings [7]. This approach can enable quick affordable access to mental health services and minimize stigma and discrimination [4].

In order to improve the detection and management of mental disorders by primary healthcare workers (PHWs) the World Health Organization (WHO) developed the mhGAP Intervention Guide (mhGAP-IG) [8]. The guide was designed to be used by non-specialists in health facilities at primary care level. It contains evidence-based interventions to identify and manage ten priority disorders and serves both as a teaching and implementation tool.

The disorders included in the guide are psychosis, alcohol and drug use, depression, bipolar disorder, dementia, developmental and behavioural disorders, medically unexplained complaints, epilepsy and suicide. The WHO recommends that the mhGAP-IG is adapted by countries to suit their local context, resources and priorities.

The impact of introducing training in the WHO mhGAP-IG for primary care staff in Malawi is unknown. The current study evaluates a mhGAP training and supervision programme by assessing knowledge, attitudes, confidence, and number of new mental health cases detected by PHWs in Mulanje district, Malawi. Mulanje acted as a pilot district for a programme of mhGAP training and supervision delivered in five districts in southern Malawi. The project was one component of a larger educational programme funded by the Scottish Government.

## Methods

### Study design

**Fig. 1:**
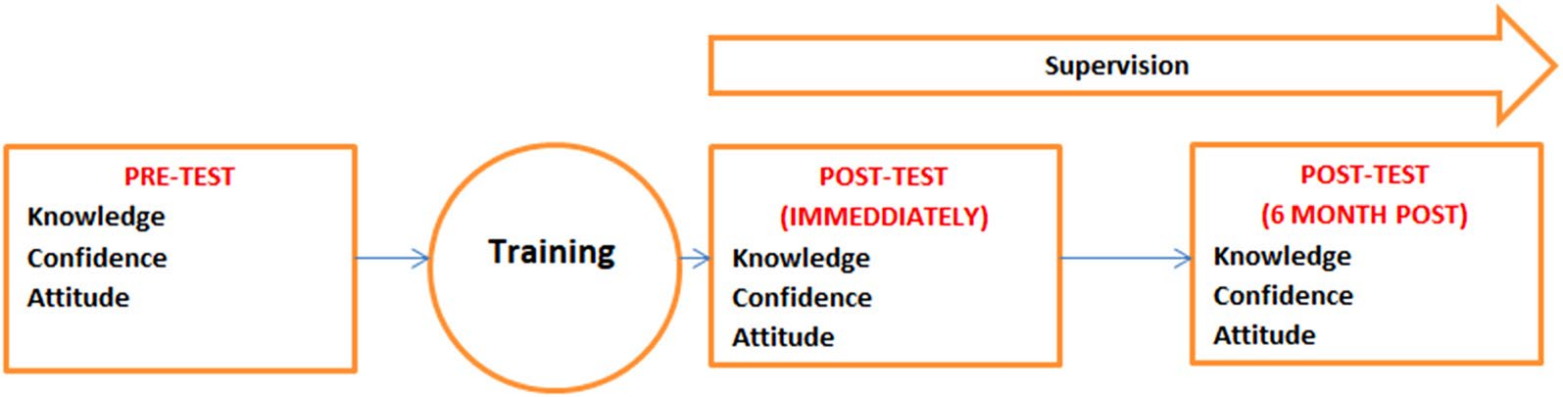
Quasi-experiment design used in the study

The study employed a quasi-experimental approach (single cohort with pre- and post- measures), with an interrupted time-series design. The implementation of mhGAP training was part of the design. Knowledge, attitudes and confidence were measured before, immediately after and 6 months after the mhGAP training (Fig. 1).

**Fig. 2:**
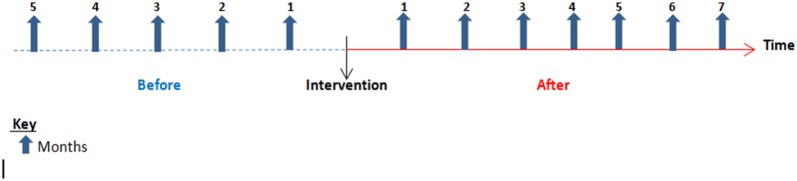
Interrupted time-series design for service utilization data collection

To assess the number of new cases detected by PHW, measurements were made every month for 5 months before and 7 months after mhGAP training (Fig. 2).

### Setting

The study was conducted in the district of Mulanje. Mulanje is in the Southern Region of Malawi close to the border of Mozambique. It has a total population of 684,107 people, 558 villages and 160,147 households [9]. The main economic activity is tea production. The poverty level recorded by the National Statistical Office is 68.6%, as defined by Foster et al. [10]. The literacy level is 60%. Life expectancy at birth for males and females was 61 and 67 respectively in 2016 [11]. The district has one government hospital, one Christian Association of Malawi (CHAM) hospital, 18 government primary care health centres, 3 dispensaries and 45 PHWs. Mulanje was chosen through an agreement with the Ministry of Health and ZMH. It was one of the districts in Malawi with high rates of patient referrals to ZMH.

### Study population and sample

The study targeted all 45 PHWs from the 18 government primary care health centres in Mulanje District. These include nurses who hold a certificate in nursing and midwifery, medical assistants hold a certificate in medical sciences, and clinical technicians with a diploma in medical science. Most health centres had two PHWs (a nurse and medical assistant) but some had three or four PHWs. The health centres were Chinyama, Chambe, Thuchira, Mimosa, Namphungo, Chonde, Chisitu, Mloza, Mpala, Naphimba, Nkomaula, Mbiza, Namasalima, Kabenje, Namulenga, Bondo, Milonde and Mlomba. Out of 45 PHWs invited, 43 attended the training and agreed to participate in the study. The other 2 were on holiday and did not show up for the training despite efforts to have them attend. During the training, corresponding names, codes and contact details of all participants were recorded to avoid loss of follow-up due to transfer, resignation or retirement.

The number of diagnosed mental health cases was obtained from clinic records for the period of 5 months prior and 7 months post training. The primary care records consisted of a standard register, which listed all patients seen, their basic demographic data, diagnosis and diagnostic codes. Before the training, mental disorders had only two codes; acute psychosis and chronic psychosis. During the training, the PHW’s were instructed to start using specific descriptions of different disorders such as depression, schizophrenia, mania and alcohol and substance abuse. These became part of the routine register.

### Training and supervision

The training package followed a ‘train the trainer’ model. A team of five health workers (consisting of a psychiatric clinical officer with a BSc in psychiatry and four registered psychiatric nurses with a diploma in psychiatry) were trained by a team of mental health professionals to deliver a mhGAP training package to all PHW’s from participating health centres. The mental health professionals comprised of a senior psychiatric clinical officer from ZMH (BSc in clinical medicine, MPhil Public Mental Health), a nurse lecturer from Malawi College of Health Sciences (BSc Mental Health & Psychiatric Nursing) and a consultant psychiatrist from the UK (BSc, MB ChB, FRCPsych).

The training was delivered using presentations, tutorials, videos, case studies and role-plays. The mhGAP package was adapted to fit the Malawian setting. For example, all training videos used were screened in the local language—Chichewa. The core conditions were moderate to severe depression, alcohol and substance misuse and psychosis. These conditions were thought to be the most important mental disorders presenting at primary health level in Malawi, following consultation with representatives in the Ministry of Health. The training package was divided into three modules and was delivered in 2 days. Module 1 and 2 were taught on day 1 and comprised of detection and management of moderate–severe depression and alcohol and drug use disorders. Module 3 was taught on day 2 and comprised of detection and management of Psychosis, rational ordering of psychiatric drugs and recording of psychiatric cases.

The training also involved on-going support and supervision through monthly outreach visit to each health Centre. This was done by the team of master trainers for the first 3 months and continued by the DMHT team using their routine supervision schedule. During a visit, the trainers observed and shadowed each PHW as they were conducting a mental health clinic. An mhGAP supervision form was used to rate each PHW. Meetings were held with the PHWs at the end of each visit to discuss their performance and make recommendations for improvement. In order to have objective assessment of PHWs performance a mental health case register was created for the participants to record patients seen throughout the previous month. This was done in order to make discussion and supervisor assessment more consistent.

### Peer support groups

In parallel with the training, was the establishment of Peer support groups comprising of mental health users and their carers. This was done with the help of Mental Health Users and Carers Association of Malawi (MeHUCA), a nationally registered patient advocacy organisation. The support groups were meant to be a platform for sharing support and experiences.

### Data collection

Prior to the start of their training, participants completed three pre-training questionnaires assessing attitudes, knowledge and confidence. Each participant was allocated a participant code which they wrote on the questionnaires to allow the pre and post-intervention questionnaires to be paired for analysis. Immediately after and 6 months after the training, participants completed the same questionnaires.

Participants were provided with verbal and written information on the study. Informed written consent was obtained from all participants. Participants were free to withdraw from the study at any time without withdrawing from the training itself. Participants were also encouraged to talk about the study with the investigators and ask any questions.

Clinical registers held at all 18 health centres in Mulanje were used to collect data on the number of new cases of mental disorders (including both description and code) detected by primary health workers in the 5 months prior (January to May 2014) and 7 months after training (June–December 2014). The first author (DK) collected this information.

### Instruments

Three self-administered questionnaires were used to collect information from participants. These were the Community Attitudes toward Mental Illness (CAMI) scale developed by Taylor and Dear [12], the WHO mhGAP pre-and-post knowledge test for mhGAP based training [13] and a confidence questionnaire [14].

The CAMI is a self-administered questionnaire that is used to measure whether an individual or a group of people hold positive or negative attitudes towards mental illness and the mentally ill. It uses a five-point Likert scale (strongly agree, agree, neutral, disagree and strongly disagree) and consists of 40 items. The CAMI is divided into four subscales namely benevolence, authoritarianism, community mental health ideology and social restrictiveness, each with 10 items. A mean score for each subscale is obtained. A score for each subscale rages from 10 to 50. The higher a mean on a particular subscale, the more of that attribute a person or group has. The CAMI has been demonstrated to be reliable and has been used in a number of previous studies in Nigeria [15], Ghana [16], and South Africa [17]. The CAMI was slightly modified to make it country specific. Words such as ‘neighbourhood’ in some items was replaced with ‘village’ since neighbourhood was seen as a western concept not equivalent to a village in Malawi. Dollar was replaced by kwacha in one item (item n) to reflect the local currency.

The WHO mhGAP pre-and-post test for mhGAP based training was used to assess knowledge. The questionnaire has 20 items related to major mental health disorders. Ten of the items are multiple choice questions while the other 10 are true and false questions. A score of 1 was assigned to every correct answer while a wrong answer got a score of 0. If a participant answered all the 20 items correctly, his/her total score was 20. The higher the score the more knowledgeable a participant was. The scores were then used to calculate mean scores.

To assess the confidence of the primary health workers, the researcher used a confidence questionnaire previously used in Malawi in a study which assessed the confidence of Health Surveillance Assistants in identifying mental disorders following a different mental health training [14] The questionnaire has 14 items rated on a Likert scale where response categories range from 1 (very confident) to 4 (not at all confident) The highest possible was 56. The higher the score, the more confident the participant was in identifying mental disorders.

### Data analysis

All variables were checked for integrity and consistency before conducting analysis using Stata version 13.1 (Stata Corp, Texas, USA). For continuous variables, summary statistics were obtained and presented either as a median (inter quartile range [IQR]) or mean (standard deviation [SD]) dependent on whether the data were normally distributed.

Given that the scores for the variables knowledge, confidence and attitudes were normally distributed, a paired t-test and one-way analysis of variance (ANOVA) was conducted using ‘before’, ‘immediately after’ and ‘6 months after’ mean score to determine any change in these measures following the training. Repeated measures ANOVA was used to account for repeat measures on the same people.

In order to determine the number of new cases detected, mean numbers of cases for each month were plotted over time using sequence line graphs disaggregated by clinic charts. We aggregated the data for all clinics and used segmented linear regression as described by Lagarde et al. [18] to determine whether there was a significant change in case detection/cases before and after the intervention. We used the Prais-Winsten Method to adjust for autocorrelation.

## Results

### Characteristics of study population

Table 1 summarizes characteristics of the study participants. Out of the 43 participants, 26 (60.5%) were male and 17 (39.5%) were female. The median age of the participants was 34 years (IQR: 30–42). Their median years of clinical experience was 5 years (IQR: 3–10) but only 3 participants (7.0%) had previous training in mental health or psychiatry after their general training. None of the study participants had any in-service training in mental health with most of the participants working either as nurse midwife technicians (19 (44.2%)) or as medical assistants (20 (46.5%)).

**Table 1:** Characteristics of the study participants

Variable	Characteristic	n	%
Gender	Male	26	60.5
	Female	17	39.5
Age (years)	Median (IQR)	34	30–42
Years of clinical experience	Median (IQR)	5	3–10
Previous training in mental health/psychiatry after generic training?	Yes	3	7.0
	No	40	93.0
In-service training in mental health?	Yes	0	0.0
	No	43	100
Current role	Nurse midwife technician	19	44.2
	Medical assistant	20	46.5
	Clinical technician	1	2.3
	Community nurse	2	4.7
	Other	1	2.3

Table 2 shows Knowledge, Confidence and Attitude test mean scores before, immediately after and 6 months post training.

**Table 2:** Knowledge, confidence and attitude test mean scores

	Pre-training	Post-training	6 month post
Knowledge	11.8	15.1	13.9
Confidence	39.9	49.6	46.8
Full CAMI	127.3	127.9	128.9

### Knowledge

The mean knowledge score increased significantly from 11.8 (SD: 0.33) before training to 15.1 (SD: 0.38) immediately after training; (t (42) = 7.79, p < 0.01). Similarly, mean knowledge scores were significantly higher 6 months after training (13.9, SD: 2.52) than before training (t (42) = 4.57, p < 0.01). ANOVA showed an overall significant difference in mean knowledge scores before, immediately after and 6 months after training (F2, 126, 0.05 = 22.1; p < 0.01).

### Confidence

The mean confidence scores increased significantly from 39.9 (SD: 7.68) before training to 49.6 (SD: 06.14) immediately after training (t (84) = 8.43, p < 0.01). Similarly, mean confidence scores were significantly higher 6 months post training (46.8, SD: 6.03) than before training (t (84) = 6.60, p < 0.01). ANOVA showed overall significant difference in mean confidence scores before, immediately after and 6 months after training (F2, 126, 0.05 = 42.7; p < 0.01).

### Attitudes

One-way analysis of variance (ANOVA) showed that there was no overall significant difference in mean CAMI scores before, immediately after and 6 months after training in all four of the CAMI components. The F-test statistic and p-value were F2, 126, 0.05 = 2.5; p = 0.09 (Authoritarianism), F2, 126, 0.05 = 0.1; p = 0.9 (Benevolence), F2, 126, 0.05 = 0.03; p = 1.0 (Social Restrictiveness) and F2, 126, 0.05 = 0.04; p = 1.0 (Community Mental Health Ideology). No pair-wise comparisons with t-tests were undertaken following the results from ANOVA. Table 3 shows CAMI sub-scales test mean scores and Standard Deviation.

**Table 3:** CAMI sub-scales test mean scores and Standard Deviation

	Before training Mean (SD)	Immediately after training Mean (SD)	Six months after training Mean (SD)
Authoritarianism	33.1 (4.49)	35.3 (4.66)	34.1 (4.72)
Benevolence	34.3 (4.51)	34.4 (4.27)	34.0 (4.16)
Social Restrictiveness	27.9 (4.83)	27.8 (5.00)	27.7 (4.23)
Community Mental Health			
Ideology	32.0 (3.92)	31.7 (4.75)	32.0 (4.69)
Full CAMI	127.3 (8.93)	127.9 (10.82)	128.9 (10.40)

### Time series analysis for new cases identification

Table 4 shows results of the segmented regression comparing the mental health service utilisation before and after the intervention.

**Table 4:** Results of the segmented regression comparing the mental health service utilisation before and after the intervention

Cases per month	β (95% Confidence Interval)	P-value
Trend prior to training	− 0.22 (− 2.67; 2.23)	0.84
Change in training month	1.63 (− 7.31; 10.57)	0.69
Trend post training	2.43 (1.02; 3.83)	< 0.01
Constant	17.27 (9.22; 25.32)	< 0.01

**Fig. 3:**
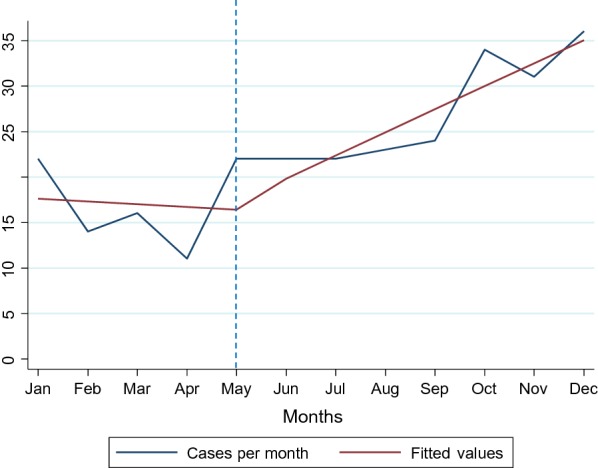
Cases per month aggregated across all facilities and the fitted values from the results of the segmented linear regression

The segmented linear regression showed that there was no increasing trend in mental health service utilization prior to the intervention (β = − 0.22 (95% CI − 2.67; 2.23)). There was no immediate significant increase in mental health service utilization in the month of training (β = 1.63 (95% CI − 7.31; 10.57)) but there was a significant positive change in the trend in mental health service utilization after the intervention (β = 2.43 (95% CI 1.02; 3.83)) (Fig. 3).

## Discussion

The findings of this study add to the growing evidence for policy makers of the effectiveness of mental health training and supervision of primary care workers in improving knowledge, confidence and case detection in a resource-constrained country. The results are consistent with other studies done in South Africa, Kenya and Malawi in which knowledge and/or confidence of primary health care workers have been found to improve significantly after a mental health training intervention [14, 19, 20]. The results are also consistent with another study in Malawi which found an increased number of diagnosed mental health cases after a training intervention [21]. Interestingly, the findings of our study show that while it is possible to improve confidence and knowledge in primary care workers, their attitudes are much more difficult to change. To the best of our knowledge, only one study from sub-Saharan Africa looked at changes in attitude of health workers following a mental health training intervention and found a significant improvement [22].

The mhGAP training may be a useful addition to developing mental health capacity in Malawi. We would need to trial it in a number of other districts to see if the results are reproducible. The following are the main strengths of the model. Firstly, the model can easily be embedded into the routine training structure of a district. The training can be delivered in 2 days and can be part of continuous professional development. Similarly, supervision can be provided through already existing structures such as outreach clinics.

Secondly, the training is sustainable. This is because it uses secondary level hospital trainers in a particular district to deliver the training package and supervision. In this way, refresher training and training of new primary health workers in a district can easily be done by the trainers. This also means that expertise stays in a particular district and there is continuous monitoring and mentoring of PHWs.

Thirdly, the training uses materials that are evidence based and adapted for use in a Malawian setting. For example, all case vignettes and role-plays had been adapted to ensure the clinical scenarios were relevant to the Malawian context. In addition the videos used were part of a series already prepared in the local language.

## Limitations

The study has a number of limitations. Firstly, the sample size was too small to confidently generalise the finding to the whole population of Malawi. Unfortunately, it was not possible to increase the sample size as we used a total enumeration of primary healthcare workers in Mulanje available at the time.

Secondly, the quasi-experimental method used to evaluate the training is susceptible to confounders. Using this method it is difficult to infer causality with the same level of confidence as in a randomized controlled trial. We tried to reduce confounders by also using a time-series design for case identification that enables multiple measurements before and after training to be made, strengthening possible causal attribution.

Thirdly, for case identification we were only able to use the total number of mental disorders per month for the 5 months before instead of comparing case detection for each disorder covered in the training package i.e. moderate-severe depression, alcohol and drug use disorder and psychosis. It was impossible to separate the individual disorders as only two codes, acute and chronic psychosis, were used in the health centres’ clinical registers prior to the intervention.

Fourthly, the study was only able to assess the changes in the number of people diagnosed but not assess whether they were accurately diagnosed.

Finally, it was also not possible to link each health care worker to the number of patients they saw. This makes it difficult to know if those health care workers who scored low on attitudes, knowledge or confidence were responsible for lower case detection rates. It may have been that increases in case detection were due to other health system factors such as increased availability of medication influencing health care workers practice.

## Conclusions

The results show improvements in the knowledge, confidence and detection of severe mental illness in primary care in Mulanje and demonstrate the potential for narrowing the treatment gap by rolling out mhGAP training nationally in Malawi. The findings of this study add to the growing evidence for policy makers of the effectiveness of mental health training and supervision of primary care workers in a resource-constrained country. Further research is needed to evaluate factors that may lead to change in health worker attitudes, to evaluate training and supervision programmes using more robust evaluation designs, such as randomised controlled trials, and to assess the feasibility and effectiveness of scale up of mhGAP programmes at larger population levels.

## Data Availability

The datasets used and analysed during the current study are available from the corresponding author on reasonable request.

## Abbreviations

ANOVA: one way analysis of variance
CAMI: Community Attitudes towards the Mentally Ill
CHAM: Christian Health Association of Malawi
HIC: high income country
IQR: interquartile range
LIC: low income Country
mhGAP: Mental Health Gap Action Programme
mhGAP-IG: Mental Health Gap Action Programme-Intervention Guide
PHW: primary healthcare worker
SD: standard deviation
WHO: World Health Organisation
ZMH: Zomba Mental Hospital
